# No evidence of ongoing evolution in replication competent latent HIV-1 in a patient followed up for two years

**DOI:** 10.1038/s41598-018-20682-w

**Published:** 2018-02-08

**Authors:** Hoi Ping Mok, Nicholas J. Norton, Jack C Hirst, Axel Fun, Mikaila Bandara, Mark R. Wills, Andrew M. L. Lever

**Affiliations:** 1 Department of Medicine, University of Cambridge, Cambridge, UK; 2 Yong Loo Lin School of Medicine, Singapore, Singapore

## Abstract

The persistence of infected T cells harbouring intact HIV proviruses is the barrier to the eradication of HIV. This reservoir is stable over long periods of time despite antiretroviral therapy. There has been controversy on whether low level viral replication is occurring at sanctuary sites periodically reseeding infected cells into the latent reservoir to account its durability. To study viral evolution in a physiologically relevant population of latent viruses, we repeatedly performed virus outgrowth assays on a stably treated HIV positive patient over two years and sequenced the reactivated latent viruses. We sought evidence of increasing sequence pairwise distances with time as evidence of ongoing viral replication. 64 reactivatable latent viral sequences were obtained over 103 weeks. We did not observe an increase in genetic distance of the sequences with the time elapsed between sampling. No evolution could be discerned in these reactivatable latent viruses. Thus, in this patient, the contribution of low-level replication to the maintenance of the latent reservoir detectable in the blood compartment is limited.

## Introduction

The principal barrier to the eradication of HIV is a reservoir of latently infected cells that serves as a source of viral recrudescence upon cessation of antiretroviral therapy (ART). The most widely studied latent virus pool is that found in circulating resting CD4^+^ T cells. This reservoir is stable despite years of effective ART; the mechanisms by which it is maintained are incompletely understood. Longitudinal studies conducted on patients under ART examining reactivatable latent viruses in children^1^, and proviral DNA together with pre-treatment plasma viral RNA in children^2^ and adults^3^, have not revealed any evidence of viral evolution in the blood compartment, suggesting that persistent viral replication does not contribute to the stability of the reservoir. However, recent findings indicate that low level ongoing viral replication occurs at sanctuary sites such as lymphoid tissue despite effective ART in some patients^4,5^. An alternative, but not mutually exclusive, hypothesis is that clonal expansion of latently infected cells is involved in the stability of the latent reservoir^6–8^.

Here we report a case study conducted over 103 weeks on an HIV positive patient diagnosed with haemochromatosis, the treatment for which demanded frequent large volume venesection. This offered an opportunity to study the evolution and clonality of replication competent latent viruses in the blood compartment. Strikingly, but consistent with previous longitudinal studies, over this two-year period no ongoing evolution was observed in this rigorously defined, physiologically relevant latent HIV reservoir.

## Methods

### Ethics

The participant gave written informed consent and this study was approved by the National Health Services (NHS) Health Research Authority (UK) under REC reference 12/SC/0679. All experimental procedures were approved by the institutional review board of the University of Cambridge and were performed in accordance with the relevant guidelines.

### Outgrowth assay

PBMCs were isolated from whole blood by gradient density centrifugation using Lymphoprep (Stemcell Technologies). CD69-CD25-HLA-DR- resting CD4 + T-cells were isolated by negative selection using a custom antibody kit (Stemcell Technologies). Cells were seeded at 0.3–0.5million cells per well and stimulated with 2 μg/ml phytohaemagglutinin, a 10 fold excess of irradiated allogenic PBMC and 10 units/ml IL-2. 0.1 or 0.5million SupT1-CCR5 feeder cells were added to each well and maintained in culture for up to 21 days^9^.

### Generation of HIV_NL4–3_ infected cells

293 T cells were transfected with the plasmid pNL4-3 by the calcium phosphate method. Virus-containing supernatant was harvested 48 hours after transfection. SupT1-CCR5 cells were infected by spinoculation. After infection, the supernatant was thoroughly washed away. Limiting dilution of infected cells was performed and mixing with uninfected SupT1-CCR5 cells such that overall number of cells seeded into each well was constant at 0.1million cells/well. Cultures showing cytopathy were expanded and maintained for a total of 21 days.

### Viral RNA extraction and sequencing

Viral RNA was extracted from virus containing supernatants of positive wells using QIAamp Viral RNA Mini Kit (Qiagen). Viral RNA was converted to cDNA using the High Capacity cDNA reverse transcription kit (Thermo Fisher). cDNA was purified (Qiagen). Gag and env amplicons were generated with the following primers: *gag* forward GGGGACATCAAGCAGCCAT; *gag* reverse CAGCCCTTTTTCCTAGGGGC; *env* forward TGTGTACCCACAGACCCCAA; *env* reverse CTTCCTGCTGCTCCCAAGAA using GoTaq DNA Polymerase (Promega). PCR was conducted with the following conditions: initial denaturation 95 °C for 2 minutes, amplification 35 cycles of 95 °C for 1 minute, 58 °C for 30 seconds, 72 °C for 90 seconds, final elongation 72 °C for 5 minutes. PCR products were purified (Qiagen). Sequencing was carried out on an ABI 3730xl DNA Analyzer (GATC and Eurofins).

### Sequence analysis

Sequence chromatograms were examined manually and base calls corrected. Sequences were aligned using ClustalΩ and alignments were manually inspected. Pairwise distance was computed using the pairwise deletion method. All ambiguous positions were removed for each sequence pair. Dendrograms were constructed based on pairwise distance data using Mega7. Linear regression was performed using Prism 5.

## Results

The HIV positive patient involved in this study had been stably treated with ART for over 7 years. The patient had experienced a ‘blip’ 6 months prior to the study, but aside from this had maintained an undetectable viral load (<50 copies/ml) from six months prior to the start of this study and through the entire sampling period (Table 1).

**Table 1:** Profile of the patient involved in the study.

60 year old male
Time since HIV diagnosis: 7 years
Time on treatment: 7 years
Nadir CD4^+^ count: 30 cells/μl
Peak viral load: 5.6 log copies/ml
Treatment regimen: Tenofovir, emtricitabine, boosted atazanavir
CD4^+^ count at start of sampling: 440 cells/μl
Viral Load at time of sampling < 50 copies/ml
Time since last viraemia: 47months
Time since last blip (viral load > 50 copies/ml on single sample): 6 months

Blood draws of 200–600 ml were obtained at 9 time points over 103 weeks. In order to determine the genetic makeup of replication competent HIV from the latent reservoir, resting CD4^+^ T cells (CD69^−^CD25^−^HLA-DR^−^) were isolated and seeded into a limiting dilution viral outgrowth assay^9^. Latent HIV was reactivated by stimulation of T cells with PHA and irradiated allogeneic PBMCs. The activated cells were co-cultured with SupT1-CCR5 feeder cells for 21 days to amplify any released virus. Wells were determined to be positive for virus outgrowth when cytopathic effect was observed by microscopy. In our experience, directly observed cytopathic effect approximates a near perfect correlation with release of viral capsid protein p24 as determined by ELISA, as reported previously^9^. In approximately half of all wells seeded with 0.5 million resting CD4^+^ T cells, virus outgrowth was observed. Thus the majority of wells at this or higher dilutions could be expected to contain a single reactivatable replication competent latent virus. Viral RNA was extracted from the supernatant of positive wells at this or higher dilutions and reverse transcribed. Amplicons were generated in *gag* and sequenced by Sanger sequencing. 64 viral sequences were obtained over the study period; these were aligned and trimmed to yield a 534 bp region of *gag* corresponding to nucleotide 1475 to 2011 of HIV strain HXB2.

To control for nucleotide mutations introduced by the virus outgrowth procedure and errors introduced by reverse transcription, amplicon generation and sequencing steps, SupT1-CCR-5 cells were infected with a laboratory virus, HIV_NL4-3,_ and a limiting dilution procedure was performed. At the dilution where cytopathic effect was observed in no more than half of the wells, the majority of the wells could be expected to have received no more than one infectious unit. These infected cells were cultured for a total of 21 days to mimic the virus outgrowth assay. Viral RNA was extracted from the supernatants of these wells and sequenced using the same method as for the patient samples. We obtained 24 control sequences. At the corresponding 534 bp region of *gag* as the patient samples all control sequences derived from the HIV_NL4-3_ cultures were identical. Thus differences in viral sequence detected in the patient samples are likely to reflect underlying diversity of the reactivated provirus rather than spontaneous mutations or errors introduced by the experimental technique.

**Figure 1:**
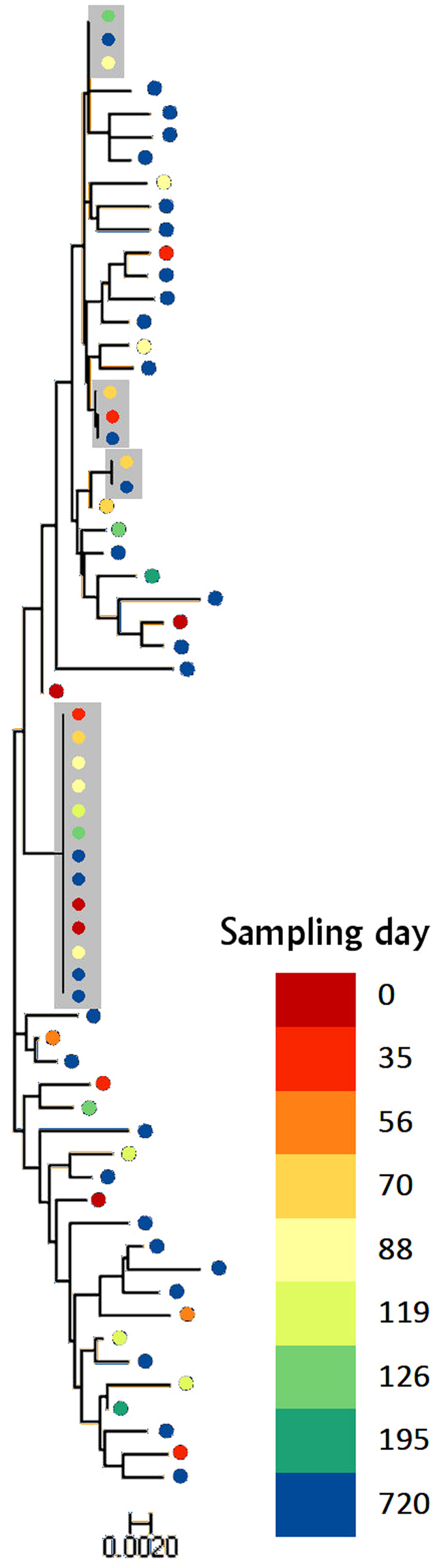
Dendrogram constructed using the neighbour joining method from 64 *gag* sequences obtained from replication competent virus activated from resting CD4^+^ T cells from the patient donor. Branch lengths as indicated by the scale bar represent the p-distance between sequence pairs. Grey boxes represent isolates which could not be distinguished using their *gag* sequence alone. The pink box highlights eight viruses with identical *gag* sequences which could not be discriminated by sequencing *env*. The dots are coloured according to the key to show date of sampling. There is no apparent clustering by time of sampling.

**Figure 2:**
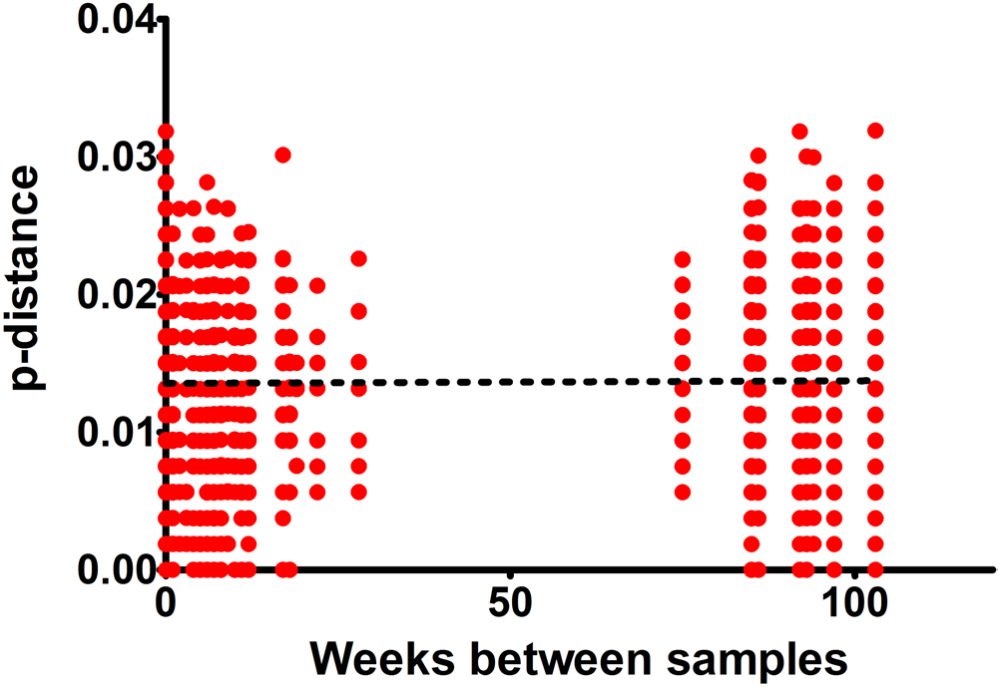
Linear regression of p-distance and time between sampling. For each pair of samples we compared the pairwise distance and the time elapsed between the samples being taken. No association between time of sampling and p-distance was identified (r^2^ = 0.00016, p = 0.573).

To detect evidence of new viruses seeding into the latent reservoir we constructed a dendrogram using the neighbour joining method, seeking any increasing sequence divergence with time. No obvious pattern with respect to time of sampling emerged (Fig. 1). To ensure that our observation had not arisen due to the method of analysis, we also analysed the sequencing data using the maximum likelihood method and maximum parsimony method and again found no evidence of clustering by time of sampling (Supplementary Figure 1). To analyse this further we used a linear regression of time between sampling and pairwise distance for each pair of sequences (Fig. 2) which provided no evidence that sequences increased in diversity the further apart in time they were sampled (p = 0.57).

From the dendrogram four clusters of identical sequences were observed. These consisted of a large group of 13 sequences, two groups of three sequences and one group of two. Sequencing of *env* was attempted on the viruses in which *gag* sequences were identical. This allowed 5 of the 13 viruses in the large cluster and all viruses in the smaller clusters to be discriminated, as pairwise differences of *env* between these apparently identical viruses are higher than that of the corresponding *env* regions in the control sequences. The remaining 8 out of 64 sequences could not be distinguished.

## Discussion

Here we report on the sequence diversity of replication competent latent HIV derived from T cells isolated over 103 weeks. We utilized resting CD4^+^ T cells so that only latently infected cells are included, and our method ensured that the only sequences included were derived from reactivatable, replication competent viruses. Whilst several recent studies have provided information on the sequences of replication competent, latent HIV^6,10^ longitudinal data remain scarce^1^.

Although the patient required regular therapeutic venesections for haemochromatosis, the condition is not known to affect HIV latency. Unlike post mortem studies, the data are free from confounders caused by terminal ill health, such as the inability of the patient to take or absorb antiretrovirals. Furthermore, our data are unaffected by potential artefacts due to co-morbidities such as malignancy.

We hypothesise that if low-level replication was responsible for the maintenance of the reservoir of latently infected resting CD4^+^ T cells, viral evolution would occur over time and viruses sampled at time points closer together should be more similar than those sampled further apart. Instead the results show no relationship between the similarity of the viral sequences and sampling time. Thus we have found no evidence of ongoing evolution in the latent reservoir in this stably treated patient. This observation is consistent with other longitudinal studies conducted on material from the blood compartment^1–3^ and is in contrast with studies which have identified ongoing evolution in tissue reservoirs^4,5^. Therefore, if ongoing replication is occurring, its contribution to the maintenance of the reservoir of latently infected resting CD4^+^ T cells in the blood compartment is limited.

It has been suggested that clonal proliferation of latently infected cells contributes to the maintenance of the latent HIV reservoir. Clonally expanded sequences have been reported in the residual viraemia or rebound viraemia of treated patients^7,11–14^. Isolation of HIV with identical sequences from the latent reservoir of infected patients was reported recently^6,15^. Possible clonal viruses can also be discerned from our data with 8 out of 64 sequences that  could not be distinguished. We did not detect a large clone of latently infected cells reminiscent of the ‘predominant plasma clone’ described in residual viraemia^13^. Thus, if homeostatic proliferation were a mechanism involved in the maintenance of the latent reservoir in this patient, each clone has proliferated only to a limited extent and constitutes only a fraction of the reservoir.

We have focused the main analysis on a region in *gag*. This was a pragmatic choice. Compared with some other regions of the viral genome, *gag* is less likely to evolve *ex vivo* during the virus amplification process. Data on conserved sequences in *gag* are available to facilitate primer design^16^, and in our experience amplicons for sequencing could be generated reliably from this region. An *in silico* analysis by Laskey *et al*. has revealed that no individual viral sub-genomic region is superior to another in predicting clonality, and the ability to predict clonality with sub-genomic sequencing varies with sample type^17^. Their analysis did not include viral sequences obtained from outgrowth assays. As we have not obtained the sequences of whole viral genomes we cannot definitively determine clonality. However, the proportion of potentially identical isolates from different wells of virus outgrowth (8/64) in our study is consistent with other studies^10^.

There are some limitations in this study. We have not sequenced the integrated proviruses and thus cannot absolutely exclude the possibility of artefacts introduced by virus outgrowth, although this would likely have increased diversity rather than led to sequence conservation. We limited our study to the blood compartment to avoid the risks of invasive sampling to the patient. We note that others have reported that HIV reservoir in the blood compartment may not recapitulate that of the tissue compartments^4^ but at least some latently infected cells in the blood are found to derive from lymphoid tissues. Finally, an important limitation is that our data is derived from a single patient. Nonetheless, carefully conducted studies based on individual patients have previously provided important insights into HIV latency^18,19^.

In contrast to the evidence of evolution of HIV in tissues over six months despite effective ART^4^, longitudinal follow up over 103 weeks has not revealed evidence of ongoing evolution of the replication competent latent HIV-1 reservoir in the blood compartment in this stably treated patient.

## Electronic supplementary material

Supplementary material

## References

[1] Ruff CT (2002). Persistence of wild-type virus and lack of temporal structure in the latent reservoir for human immunodeficiency virus type 1 in pediatric patients with extensive antiretroviral exposure. J. Virol.

[2] Van Zyl, G. U. *et al*. No evidence of HIV replication in children on antiretroviral therapy. in *Journal of Clinical Investigation*10.1172/JCI94582 (2017). 10.1172/JCI94582 PMC5617669 28891813

[3] Brodin, J. *et al*. Establishment and stability of the latent HIV-1 DNA reservoir. *Elife*10.7554/eLife.18889 (2016). 10.7554/eLife.18889 PMC5201419 27855060

[4] Lorenzo-Redondo, R. *et al*. Persistent HIV-1 replication maintains the tissue reservoir during therapy. *Nature* **530**, (2016). 10.1038/nature16933 PMC4865637 26814962

[5] Rose R (2016). HIV Maintains an Evolving and Dispersed Population in Multiple Tissues during Suppressive Combined Antiretroviral Therapy in Individuals with Cancer. J. Virol..

[6] Lee, G. Q. *et al*. Clonal expansion of genome-intact HIV-1 in functionally polarized Th1 CD4+ T cells. *J. Clin. Invest*. 10.1172/JCI93289 (2017). 10.1172/JCI93289 PMC5490740 28628034

[7] Anderson JA (2011). Clonal sequences recovered from plasma from patients with residual HIV-1 viremia and on intensified antiretroviral therapy are identical to replicating viral RNAs recovered from circulating resting CD4+ T cells. J. Virol..

[8] Hosmane, N. N. *et al*. Proliferation of latently infected CD4+ T cells carrying replication-competent HIV-1: Potential role in latent reservoir dynamics. *J. Exp. Med*. (2017). 10.1084/jem.20170193 PMC5379987 28341641

[9] Fun A, Mok HP, Wills MR, Lever AM (2017). A highly reproducible quantitative viral outgrowth assay for the measurement of the replication-competent latent HIV-1 reservoir. Sci. Rep..

[10] Bui JK (2017). *Ex vivo* activation of CD4 + T-cells from donors on suppressive ART can lead to sustained production of infectious HIV-1 from a subset of infected cells. PLOS Pathog..

[11] Kearney, M. F. *et al*. Origin of Rebound Plasma HIV Includes Cells with Identical Proviruses That Are Transcriptionally Active before Stopping of Antiretroviral Therapy. *J. Virol*. **90**, (2016). 10.1128/JVI.02139-15 PMC4719635 26581989

[12] Brennan TP (2009). Analysis of human immunodeficiency virus type 1 viremia and provirus in resting CD4+ T cells reveals a novel source of residual viremia in patients on antiretroviral therapy. J. Virol..

[13] Bailey JR (2006). Residual human immunodeficiency virus type 1 viremia in some patients on antiretroviral therapy is dominated by a small number of invariant clones rarely found in circulating CD4 + T cells. J. Virol..

[14] Kearney MF (2014). Lack of detectable HIV-1 molecular evolution during suppressive antiretroviral therapy. PLoS Pathog..

[15] Bui JK (2017). Proviruses with identical sequences comprise a large fraction of the replication-competent HIV reservoir. PLOS Pathog..

[16] Li, G. *et al*. Functional conservation of HIV-1 Gag: implications for rational drug design. *Retrovirology*10.1186/1742-4690-10-1261742-4690-10-126 (2013). 10.1186/1742-4690-10-126 PMC4228425 24176092

[17] Laskey, S. B., Pohlmeyer, C. W., Bruner, K. M. & Siliciano, R. F. Evaluating Clonal Expansion of HIV-Infected Cells: Optimization of PCR Strategies to Predict Clonality. *PLoS Pathog*. **12**, (2016). 10.1371/journal.ppat.1005689 PMC4975415 27494508

[18] Simonetti, F. R. *et al*. Clonally expanded CD4+ T cells can produce infectious HIV-1 *in vivo*. *Proc. Natl. Acad. Sci. USA*. **113**, (2016). 10.1073/pnas.1522675113 PMC4763755 26858442

[19] Hütter G (2009). Long-Term Control of HIV by CCR5 Delta32/Delta32 Stem-Cell Transplantation. N. Engl. J. Med..

