# Supplementary material for: No evidence of ongoing evolution in replication competent latent HIV-1 in a patient followed up for two years
Source: Sci Rep. 2018 Feb 8;8:2639. doi: 10.1038/s41598-018-20682-w (PMC5805784; doi:10.1038/s41598-018-20682-w)
Supplement: Supplementary file 1 — Supplementary material [file 41598_2018_20682_MOESM1_ESM.pdf]

Supplementary material

No evidence of ongoing evolution in replication competent latent HIV-1 in a patient followed up for two years

Hoi Ping MOK[1], Nicholas J NORTON[1], Jack C HIRST[1], Axel FUN[1], Mikaila BANDARA[1], Mark R WILLS[1], Andrew ML LEVER[1,2]*

Affiliations; [1] Department of Medicine, University of Cambridge, Cambridge, UK. [2] Yong Loo Lin School of Medicine, Singapore, Singapore

*Corresponding author. Mailing address: Department of Medicine, Level 5, Box 157, Addenbrooke's Hospital, Hills Road, Cambridge, UK, CB2 0QQ, Telephone: +44 (0)1223 330191
E-mail: amll1@medschl.cam.ac.uk

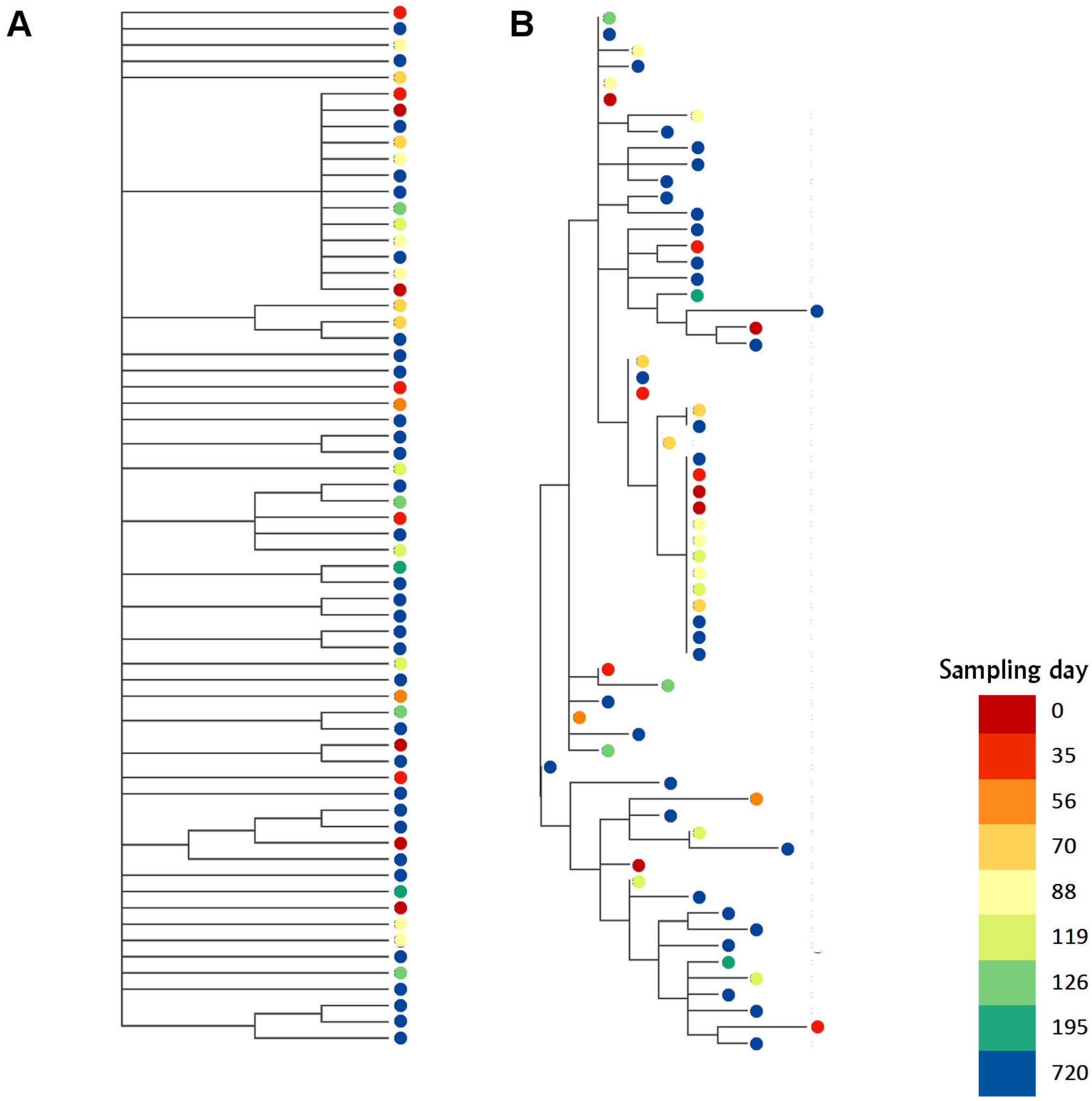

Supplementary figure 1

A. Maximum parsimony tree of *gag* sequences from reactivated patient viruses. The consensus tree inferred from 3 most parsimonious trees is shown. B. Maximum likelihood tree of *gag* sequences from reactivated patient viruses. Branch lengths show the number of substitutions per site.
